# Diabetes and COVID-19 in Congolese patients

**DOI:** 10.4314/ahs.v21i3.18

**Published:** 2021-09

**Authors:** Henriette Poaty, Guy Emergence Poaty, Gilbert NDziessi, Emile Godefroy Ngakeni, Tatiana Doukaga Makouka, René Soussa Gadoua, Regis Ondzé, Lambert Kitembo, Presley Jeanel Msengui, Jethro Massala Peya, Michel Gbala Sapoulou, Pascal Ibata, Henri Germain Monabeka, Alexis Elira Dokekias

**Affiliations:** 1 Faculty of Health Sciences, University Marien Ngouabi, Brazzaville, Congo; 2 National Research Institute on Health Sciences, Brazzaville, Congo; 3 Ministry of Public Health, Brazzaville, Congo; 4 Army Hospital, Brazzaville Congo; 5 University Hospital Center, Brazzaville, Congo; 6 Talangaï Hospital, Brazzaville, Congo

**Keywords:** COVID-19, SARS-CoV-2, diabetes, Congolese patients

## Abstract

**Background:**

The global pandemic Coronavirus Disease 2019 (COVID-19) due to the Severe Acute Respiratory Syndrome Coronavirus 2 (SARS-CoV-2) is reported to be potentially severe in patients with morbid conditions. One common reported comorbidities is diabetes. We aimed in this study to precise the clinical characteristics and outcomes in a series of congolese diabetic patients affected by COVID-19 infection.

**Patients and methods:**

We retrospectely studied from 256 COVID-19 patients, a cohort of 30 persons with previously known diabetes. The glycaemia controls have been obtained by plasma glucose assay. All patients have been tested positive to SARS-CoV-2 by RT-PCR method.

**Results:**

The COVID-19 diabetic patients represented 11,7% of all COVID-19 patients with confidence interval of 95% [7,77–15,65]. Older individuals and male sex were predominent. Dyspnea and sauration of oxygen < 90 were significatives and added risk factors were noted in 63.3% of patients, particulary hyperglycaemia with hypertension or obesity. The mortality rate at the percentage of 36.7% was more prevalent in patients with added comorbidities (30%) versus without comorbidities (6.7%).

**Conclusion:**

Congolese COVID-19 diabetic patients of male sex and older age exhibiting arterial hypertension and obesity are the most exposed to severe COVID-19 and increasead mortality rate.

## Introduction

In December 2019, cases of Coronavirus Disease 2019 (COVID-19) in the form of pneumonia due to the Severe Acute Respiratory Syndrome-Coronavirus-2 (SARS-CoV-2) was first identified in Wuhan, Hubei Province, China^1^, ^2^. On March 11, 2020, Word Health Organization (WHO) declared COVID-19 a global pandemic^3^. At the date of August 30, 2020, more than 25 million cases were reported in the world.

In Congo Brazzaville, the first case was declared on March 14, 2020. At the date of August 30, 2020, our database showed 4628 patients with laboratory confirmed COVID-19 of which 81 deaths were reported (SitRep 90, Congo).

According the review, 80% of people are asymptomatic, and approximately 20 % have symtoms of COVID-19, mainly, in cases of older persons and comorbidities as diabetes, cancer, chronic pulmonary disease, chronic renal diseases, and cardiovascular diseases^1^, ^3^, ^4^.

When SARS-CoV-2 infection occurs in people with pre-existing diabetes (type 1 and 2), there is a great risk of severe COVID-19^3^, ^5^, ^6^. The present study aimed to access to the clinical characteristics and outcomes of a series of congolese diabetic patients with COVID-19 infection.

## Patients and methods

### Patients

We retrospectely studied a clinical serie of 30 patients infected by SARS-CoV-2 with pre-existing diabetes (type 1 and type 2). They were hospitalized at two medical care sites in Brazzaville (Congo): Leyono and University Hospital Center between March and August, 30, 2020. At this point date, the cumulation of the all COVID-19 patients hospitalized, was 256 (data from hospital registries and care commission data).

## Methods

The clinical data were collected at hospital admission and during hospitalisation. COVID-19 has been classified into: 15 moderate forms (oxygen saturation > 90% or not admitted in intensive care unit) and 15 severe forms (oxygen saturation < 90% or admitted in intensive care unit).

The laboratory confirmed SARS-CoV-2 for all patients have been done by two successive real-time reverse transcriptase polymerase chain reaction (RT-PCR) assays of oropharyngeal swab specimen at National Public Health Laboratory of Brazzaville.

The glycaemia control of plasma glucose levels have been done before hospital admission, at the time of hospitalisation and during the treatment in hospital.

Statistical analysis, were conducted with epi-info 7.2.2.6. (CDC Atlanta, USA, 2017). We used chi2 Pearson test or chi2 Fischer exact to compare the proportions and Student test for the comparaison of the mean quantative data. A predictive value (p) < 5% (0.05) and 95% confidence interval were considered.

The study obtained the ethic committee approval of both hospitals and informed consent was waived by the National Technic Committee against COVID-19 in Congo Brazzaville.

## Results

### Prevalence of diabetes in COVID-19

Among a total of 256 COVID-19 infected patients, hospitalized in the two care sites, we identified 30 diabetic persons i.e. 11.7% of COVID-19 patients with confidence interval (CI) of 95% [7.77–15.65].

### Sex and age

The proportion of COVID-19 diabetic patients was 70% (21 of 30) in male gender, while it represented 30% (9 of 30) in female gender (Table 1).

**Table 1:** Clinical characteristics and outcomes of congolese diabetic patients with COVID-19

Parameters	Number (%) n=30	Outcomes (%) n=30		*p*-Value
		Survival 63,3 (11)	Death 36,7 (19)	
**Mean age (extreme), years**	56.6 (34–79)	52,74 ± 11,18	63,18 ± 11,86	**0,023**
**Sex**				0,419
Male	21 (70.00)	12 (57.14)	9 (42,86)	
Female	9 (30,00)	7 (77.78)	2 (22.22)	
**Symptoms at hospital admission**				
Fever (> 37.5)	26 (86.67)	15 (57.69)	11 (42,31)	0,267
Dry cough	25 (83.33)	14 (56.00)	11 (44,00)	0,129
Asthenia	25 (83.33)	15 (60.00)	10 (40,00)	0,626
Dyspnea	21 (70)	10 (47,62)	11 (52,38)	**0,021**
Oxygen saturation < 90 %	15 (50)	6 (40,00)	9 (60,00)	**0,021**
Arthromyalgia	6 (20.00)	3 (50,00)	3 (50,00)	0,641
Neurologic disorders (consciousness disorders, hallucinations, convulsions)	5 (16,67)	2 (40,00)	3 (60,00)	0,327
Cerebrovascular disorders (functional impotence of upper limbs, hemiplegia)	4 (13.33)	0 (0,00)	4 (100,00)	**0,012**
Headache	4 (13.3)	2 (50,00)	2 (50,00)	0,611
Nausea -Vomiting	4 (13.3)	4 (100,00)	0 (0,00)	0,268
Diarrhea	3 (10)	3 (100,00)	0 (0,00)	0,279
Sore throats	2 (6,67)	2 (100,00)	0 (0,00)	0,519
Coma	2 (6.6)	0 (0,00)	2 (100,00)	0,126
Smell and taste disorders (anosmia and dysguesia)	1 (3.3)	1 (100,00)	0 (0,00)	1,000
**Mean glycaemia** (>1.20 g/l)	1,96 ± 1,24	1,47 ± 0,78	2,82 ± 1,45	**0,013**
**Other comorbidities**				
Hypertension	15 (50)	10 (66,67)	5 (33,33)	0,705
Obesity	4 (13.3)	2 (50,00)	2 (50,00)	0,611
Chronic renal failure	1 (3.3)	0 (0,00)	1 (100,00)	0,367
Hypothyroidism	1 (3.3)	1 (100,00)	0 (0,00)	1,000
Chronic obstructive pulmonary disease	1 (3.3)	0 (0,00)	1 (100,00)	0,367
Malignancy (gastric cancer)	1 (3.3)	0 (0,00)	1 (100,00)	0,367
**COVID Diabetic patients**				0,139
with comorbidities	19 (63,33)	10 (52,63)	9 (47,37)	
without comorbidities	11 (36,67)	9 (81,82)	2 (18,18)	

**Figure 1:**
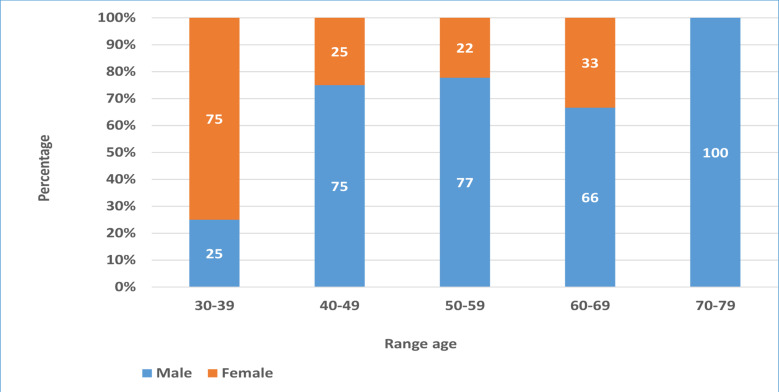
Distribution of diabetic patients with COVID-19, according age and sex.

The mean age was 56.6 years with the extreme of 34–79 years. The most concerned age range between 50–59 years for male and 30–39 years for female (Table 1, Figure 1). The mean age in survival patients was 52.74 ± 11.18 years and 63.18 ± 11.86 years in death patients (p=0.023).

### Symptoms

Fever was observed in 86.67% (26 of 30) of covid-19 diabetic, followed by dry cough and asthenia with percentage of 83.3 (25 of 30). We noted dyspnea,70% (21 of 30) (p= 0,021) and oxygen saturation was less 90 % in 50% (15/30) (p= 0,021). Other signs were: sore throats, coma, smell and taste disorders (Table 1).

### Hperglycaemia and other comorbidities

**Figure 2:**
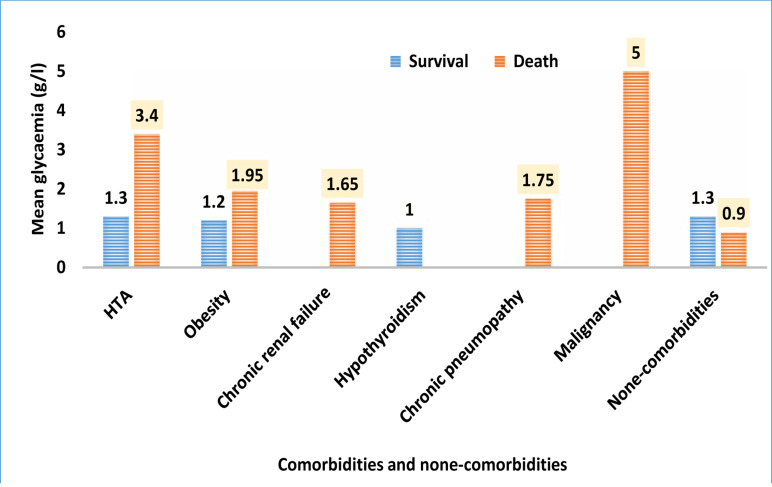
Glycaemia of COVID-19 diabetic patients with added and none added comorbidities.

The mean glycaemia was 1,96 g/l ± 1,24 (p = 0.013). In death patients, it was 2,82 ± 1,45 and in the survival patients 1.47 g/l ± 0.78. Details are reported in table 1. We noted six types of additional comorbidities (Table 1, Figure 2) from which, 50% with arterial hypertension (CI 95% [32.1–67.9]). Obesity was observed in 13.3% (CI 95% [11.49–25.45]). The mean glycaemia in case of hypertension was 3.71 g/l ± 0.68 in death patients, while it was 1.3 g/l ± 0.45 in survival patients (p = 0.000). In patients with obesity, we note a mean glycaemia of 1.95 ± 0.23 in death patients and 1.65 g/l ± 0.22 in survival patients (p = 0.072).

### Mortality

The mortality rate was 36.7% (11 of 30) of patients with diabetes and COVID-19, i.e. 4.29% (11/256) of all COVID-19 patients hospitalized during the study period (Table 1). It was 42.86% in male sex and 22% in female sex. The proportion of deaths was 47.4% in COVID-19 patients with comorbidities and 18.2% in patients without comorbidities. All of them died in respiratoy distress picture.

### Commentary

#### Pathogeny

**Figure 3:**
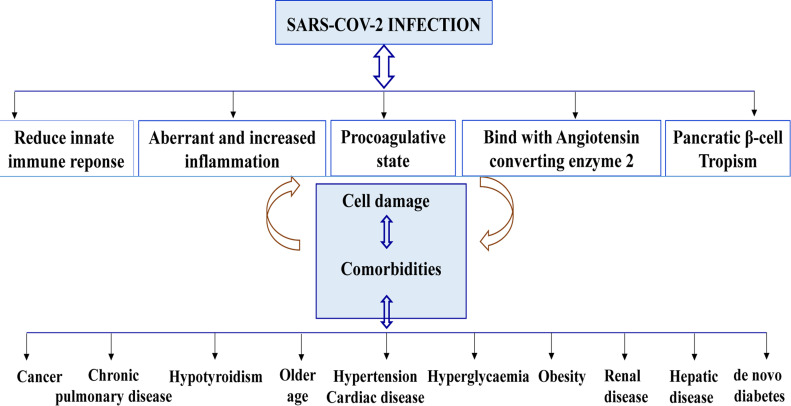
Interaction between SAR-CoV-2 infection and morbid conditions^1^, ^3^, ^7^, ^8^, ^9^, ^10^

Firstly, diabetes itself is an immune and chronic disease which predispose to morbid conditions as infections (viral, bacterial, fungal), cardio-cerebrovascular, renal and hepatic diseases. These diseases contribute to high risk to develop severe COVID-19 infection^3^. Secondly, diabetes is a comorbidity condition for COVID-19 infection. At the time of current knowleges, many viral biologic mechanisms of SARS-CoV-2 (Figure 3) are indexed to cloud the clinical picture and lead to the poor prognosis in COVID-19 patients with comorbidities. Among those mechanisms^1^.

i) Reduce innate immune reponse, because SARSCoV-2 infects alveloar cells in lungs and prevents the alveolar-capillary unit from fonctionning properly and results in respiratory dysfunction. It also infects circulating immune cells (CD3, CD4, CD8, T cells) and leads to lymphocytopenia resulting in reduction or suppression of the innate immune reponse to COVID-19 infection^3^.

ii) Aberant or increased inflammation, is due to the over production of pro-inflammatory cytokines as interleukines (IL)-1, IL-6, IL-8, tumor necrosis factor α (TNFα) and chemokines with increased blood concentration of inflammatory markers (C-reactive protein, procalcitonin, fibrinogen, ferritin). Hyperglyceamia leads also to severe inflammatory response^7^, ^8^.

iii) Increased coagulation activity, is caused by endothelial dysfunction and hypoxia that expose to thromboembolic complications^1^, ^3^. Increased inflammation and coagulative state lead to multi-organs failure resulting in severe covid and an incresead risk of death^3^, ^7^.

iv) Angiotensin converting enzyme 2 (ACE-2), is an integral membrane glycoprotein expressed on the surfaces of the epithelial cells of lungs, blood vessels, brain, cardiovascular, renal, intestinal and pancreatic tissues, and immune cells^1^, ^7^, ^9^. In normal state, ACE2 converts angiotensin II to angiotensin 1–7 and the system have antiflammatory and antioxidant role. This biologic process is disturbed in diabetes with an imbalance of ACE-2 activation pathways in case of COVID-19 infection^10^. Indeed, SARS-CoV-2 use ACE2 as a functional receptor for its spike glycoprotein (gene S), to infect epithelial cells of various organs including pancreatic β-cells^2^, ^9^, ^10^.

v) Tropism of SARS-CoV-2 for pancreatic β-cell, induces and increases insulin secretion, cell destruction resulting in additional dysregulation of glucose, worse hyperglycaemia and in some cases the onset of diabetes^3^, ^10^.

### Epidemiological data

#### Prevalence

In our study, the prevalence of persons with diabetes in the overall-COVID-19 patients was not negligible (11.7%, 95% CI 7.77–15.65%). This result is in accordance with other published data. Indeed, in one Chinese meta-analysis study including a cohort of 1527 patients with COVID-19, the prevalence of diabetes was 9.7%^3^. Recently, another china study reported a percentage of 17.4% (59) of pre-existing diabetic in a cohort of 339 COVID-19^11^. In fact, this prevalence is variable by countries, lifestyle and environment. It is globally from 8.2% to 39.5%^4^, ^12^, ^13^.

Association COVID-19 and diabetes is frequently reported. But according published papers, the prevalence is similar in general population except that: as diabetes is a morbid condition for COVID-19, it increases the risk of metabolic complications which exposes to severe COVID-19^14^, ^15^. In fact, diabetes and COVID-19 reciprocally and worsely interact and increase the mortaliy rate^3^.

### Sex and age

SARS-CoV-2 infection was higher in male gender than in female gender, sex ratio was 2.3.

About age, prevalent age ranged the older individuals (50–59 years) in male sex than in female sex (30–39 years). The difference of means between survival age and death age of the patients was significative and in accordance with previous published studies^3^. The review data suggests a link between COVID-19 and the older age of diabetic patients^14^, ^15^.

### Mortality

The mortality rate of COVID diabetic patients was higher (36.7%, 95% CI 19.45–53.95 %). Death occurred most in male sex, in older age and it was most prevalent in patients with high hyperglycaemia and in added comorbidities (Tableau 1, Figure 2). These observations are reported in several previous studies^2^, ^5^, ^13^, ^15^.

### Symptoms

The results revealad that the most common clinical signs were fever, dry cough, asthenia and dyspnea. In fact, the clinical spectrum not specific to diabetes, was variable, including several types of disorders in multi-organs (respiratory, digestive, cerebrovascular, cardiovascular and locomotive) (Tableau 1). The severity of signs were correlated with the clinical form and the presence of added comorbidities. Those clinical manifestations are also reported in several studies^4^, ^12^, ^13^.

### Hyperglycaemia and other comorbidities

Hyperglycaemia was one of the worse factor risk in our series. The mean glycaemia was significantly higher especially in persons with comorbidities, particulary arterial hypertension, obesity and one malignancy. The difference of mean glycaemia in patients with comorbid conditions and outcome was significative. High plasma glucose level was correlated with poor prognosis and mortality rate (Tableau 1, Figure 2).

Concerning the comorbidities, 63.3% of our patients had one or more comorbidities and the most common in half patients was hypertension (50%, 95% CI 32.1–67.9%), followed by obesity (13.3%, 95% CI 11.49–25.45%). The proportions of severe form of COVID-19 and deaths were higher in patients with addtional comorbidities. All these findings are in accordance with several published studies^1^, ^9^, ^13^, ^14^. Indeed, the common comorbidities reported in COVID-19 diabetic patients are: obesity, arterial hypertension, cancer, cardiovascular (myocarditis, pericardities, thromboembolic events), respiratory and cerebrovascular disesases^6^, ^8^, ^12^. All these coexisting morbid conditions are present in our cohort. They are indexed to contribute in the failure of pulmonary ventilation, electrolytes and innate immune reponse whose expose to severe COVID-19 and worse outcomes^6^.

Note that according Lei Fang^9^, diabetic patients with arterial hypertension treated with ACE inhibitors and angiotensin II type 1 receptor blockers have an increased expression of ACE that expose more to COVID-19.

## Conclusion

Congolese COVID-19 diabetic patients exhibiting hyperglycaemia, with arterial hypertension or obesity especially in older age and male gender have a higher risk of severe COVID-19, a poor prognosis and increased mortality rate. Regular glycaemia controls, research of additional comorbidities and correct management of diabetes are essential to reduce complications and mortality rate in cases of COVID-19 infection.

## References

[1] Hussain Akhtar, Bhowmik Bishwajit, do Vale Moreira Nayla Cristina (2020). COVID-19 and diabetes: Knowledge in progress. Diabetes Res Clin Pract.

[2] Pal Rimesh, Bhansali Anil (2020). COVID-19, diabetes mellitus and ACE2: The conundrum. Diabetes Res Clin Pract.

[3] Apicella Matteo, Campopiano Maria Cristina, Mantuano Michele, Mazoni Laura, Coppelli Alberto, Del Prato Stefano (2020). COVID-19 in people with diabetes: understanding the reasons for worse outcomes. Lancet Diabetes Endocrinol.

[4] Guan W-J, Liang W-H, Zhao Y, Liang H-R, Chen Z-S (2020). Comorbidity and its impact on 1590 patients with COVID-19 in China: a nationwide analysis. Eur Respir J.

[5] Peric Slobodan, Stulnig Thomas M (2020). Diabetes and COVID-19. Disease-Management-People. Wien Klin Wochenschr.

[6] Orioli Laura, Hermans Michel P, Preumont Vanessa, Loumaye Audrey, Thissen Jean-Paul, Alexopoulou Orsalia, Furnica Raluca, Burlacu Maria-Cristina, Maiter Dominique, Yombi Jean-Cyr, Vandeleene Bernard (2020). COVID-19 et diabète. Louvain Med.

[7] Huang Ian, Lim Michael Anthonius, Pranata Raymond (2020). Diabetes mellitus is associated with increased mortality and severity of disease in COVID-19 pneumonia-A systematic review, metaanalysis, and meta-regression. Diabetes Metab Syndr.

[8] Tadic Marijana, Cuspidi Cesare, Sala Carla (2020). COVID-19 and diabetes: Is there enough evidence?. J Clin Hypertens (Greenwich).

[9] Fang Lei (2020). www.thelancet.com/respiratory.

[10] Cuschieria Sarah, Grechb Stephan (2020). COVID-19 and diabetes: The why, the what and the how. Journal of Diabetes and Its Complications.

[11] Targher G, Mantovani A, Wang X-B, Yan H-D, Sun Q-F (2020). Patients with diabetes are at higher risk for severe illness from COVID-19. Diabetes Metab.

[12] Palaiodimos Leonidas, Kokkinidis Damianos G, Li Weijia, Karamanis Dimitrios, Ognibene Jennifer (2020). Severe obesity, increasing age and male sex are independently associated with worse in-hospital outcomes, and higher in-hospital mortality, in a cohort of patients with COVID-19 in the Bronx, New York. Metabolism Clinical and Experimental.

[13] Akbariqomi Mostafa, Hosseini Mahboobeh Sadat, Rashidiani Jamal, Sedighian Hamid, Biganeh Hossein, Heidari Reza (2020). Clinical characteristics and outcome of hospitalized COVID-19 patients with diabetes: A single-center, retrospective study in Iran. Diabetes Res Clin Pract.

[14] Yan Y, Yang Y, Wang F, Ren H, Zhang S, Shi X (2020). Clinical characteristics and outcomes of patients with severe covid-19 with diabetes. BMJ Open Diab Res Care.

[15] Pinto Lana C, Bertoluci Marcello C (2020). Type 2 diabetes as a major risk factor for COVID-19 severity: a meta-analysis. Arch Endocrinol Metab.

